# Real-World Performance Measurement of Patient-Centered Clinical Decision Support Tools: Qualitative Study

**DOI:** 10.2196/90000

**Published:** 2026-09-16

**Authors:** Prashila Dullabh, Courtney Zott, Nicole Gauthreaux, Abigail Aronoff, Dean F Sittig

**Affiliations:** 1 Health Sciences Department NORC at the University of Chicago Washington, DC United States; 2 Informatics Review LLC Lake Oswego, OR United States

**Keywords:** patient-centered clinical decision support, measurement, evaluation, clinical decision support

## Abstract

**Background:**

Patient-centered clinical decision support (PC CDS) includes digital technology designed to give patients, caregivers, and clinicians evidence-based, patient-specific clinical guidance to inform care decisions. PC CDS interventions cover a range of use cases, but gaps in measurement make it difficult to assess the impact of these technologies on patient and clinician decision-making, care processes, and outcomes.

**Objective:**

This study aimed to identify common measurement areas used in PC CDS projects, as well as measurement gaps, and to develop recommendations for future advancement of PC CDS measurement.

**Methods:**

We conducted a systematic review of 20 exemplary PC CDS projects funded by the Agency for Healthcare Research and Quality’s Digital Healthcare Research Program using predefined inclusion criteria. We reviewed published project materials (n=40) to gather information on the type of data, technology, and measures used, as well as conditions and populations addressed. Next, we identified a purposive sample of 9 projects and conducted key informant interviews with the principal investigators to gather perspectives on PC CDS performance measures and measurement-related limitations and challenges. We conducted a qualitative thematic synthesis to identify key themes from the reviewed material and interviews related to commonly used measures and measurement gaps in relation to a PC CDS performance measurement framework. We also gathered feedback on findings from a seven-member technical expert committee.

**Results:**

Overall, usability was the most common area of measurement across PC CDS design, development, implementation, and use phases. Projects focusing on designing and developing PC CDS technology also frequently measured acceptability, while projects focusing on implementation and evaluation frequently measured patient health outcomes, patient engagement, and clinician performance. Informants reported challenges with measuring the safety, timeliness, and cost of PC CDS technology. They also expressed the need for new measurement approaches to capture long-term health outcomes, benchmarks for meaningful patient engagement, and perspectives from people with limited digital or health literacy.

**Conclusions:**

Project investigators are using numerous measures to assess real-world PC CDS interventions, yet there are several measurement areas that need more development. The findings revealed six important next steps for future development of PC CDS performance measurement that can significantly advance the use of technologies that promote patient-centered care: (1) tracking technical performance postimplementation, (2) evaluating interventions in lower-resourced health care settings, (3) conducting more cost assessments to inform scalability, (4) streamlining privacy and security management for external data storage, (5) refining and developing standardized patient engagement measures, and (6) adapting measurement tools to populations with limited English proficiency or digital and/or health literacy.

## Introduction

Patient-centered clinical decision support (PC CDS) technologies are designed to give patients, caregivers, and clinicians evidence-based, patient-specific clinical guidance to inform care decisions [1]. PC CDS includes digital technologies that (1) incorporate evidence-based knowledge from patient-centered outcomes research (PCOR) or comparative effectiveness research; (2) incorporate patient-generated health data (PGHD), patient-reported outcomes (PROs), patient preferences, health-related social needs (HRSN), and other patient-specific information; (3) facilitate bidirectional information exchange in support of patient-centered care, including shared decision-making; and (4) are delivered directly to patients and/or caregivers via apps or portals [2].

Significant resources have been invested in PC CDS technology since the early 2010s. The establishment of the Patient-Centered Outcomes Research Trust Fund under the Affordable Care Act provided funding to support the development and dissemination of evidence at the point of care. Multiple federal agencies, including the Agency for Healthcare Research and Quality (AHRQ), have played key roles in advancing PC CDS through funding, infrastructure development, and policy initiatives [3-6].

More than a decade into this investment, PC CDS remains an emerging field, yet its continued technological advancement and implementation in clinical settings underscores a need for systematic evaluation [7]. As PC CDS technologies become more integrated into care delivery, it is critical to assess their real-world use, effectiveness, and impact on clinical and patient-centered outcomes. Measurement is essential in determining whether PC CDS interventions are achieving intended goals, as well as supporting continuous improvement of these technologies. Our research has found that existing evaluations of PC CDS have largely focused on implementation feasibility and acceptability, while providing less evidence on patient and clinician engagement or clinical outcomes. When engagement has been measured, assessments have focused on more basic measures of technology use rather than the quality or meaningfulness of the engagement, making it difficult to understand how patients and clinicians interact with PC CDS and which engagement strategies contribute to better outcomes [2,8]. Similarly, studies incorporating clinical outcomes have tended to focus on care processes (eg, adherence to a treatment plan) rather than downstream outcomes [2]. Without such understanding, the adoption and sustained use of PC CDS technology and improved clinical outcomes will remain limited.

The objective of this review was to examine how PC CDS interventions are being measured in real-world settings, identify common challenges and gaps in evaluation practices, and develop an action plan to guide future PC CDS measurement efforts. These findings will provide insight into areas for improvement of PC CDS performance measurement and contribute to the development of measures and strategies to overcome the salient challenges highlighted in the literature.

## Methods

### Study Design

We used a qualitative case study approach to examine PC CDS measurement within the real-world context of funded research projects and their investigators, allowing for in-depth exploration of processes, challenges, and contextual factors [9]. We used four methods for this case study approach: (1) systematic review of relevant projects funded by the AHRQ Digital Healthcare Research Program, (2) document review of project artifacts, (3) semistructured key informant interviews, and (4) expert review via a technical expert panel (TEP).

### Project Selection

We conducted a systematic review of PC CDS projects funded by the AHRQ Digital Healthcare Research Program [10,11]. This program funds research across the United States to advance the evidence base for digital health care and represents one of the most comprehensive federal portfolios dedicated to advancing PC CDS in real-world settings.

The inclusion criteria included projects that (1) were completed by 2024; (2) had at least one published artifact, such as a final report, manuscript, or presentation; (3) involved the use of patient-contributed data, such as PGHD, PROs, patient preferences, and/or HRSN data by a PC CDS technology [12]; and (4) included an evaluation component in the project. Projects were excluded if they did not meet one or more of the inclusion criteria.

Using project descriptions available on the AHRQ’s Digital Healthcare Research website, two independent researchers (authors CZ and NG) screened titles and abstracts of 67 projects, ultimately excluding 41 projects that did not meet the inclusion criteria. This was followed by a full-text review of publicly available documents for the remaining projects (n=26) that were linked on the website, including final reports, publications, and public webinars. Discrepancies at each review stage were resolved through consensus discussions, with arbitration by a third independent reviewer (author PD), when required. A total of 20 projects were ultimately selected for inclusion in the review, with a total of 40 associated publications. The selected projects provide a range of perspectives across PC CDS technologies, populations, and medical conditions, supporting applicability of findings to other health systems or settings.

### Document Review and Data Extraction

Two researchers (CZ and NG) independently performed data extraction using a standardized data abstraction form in Microsoft Excel that collected information on the following criteria: project characteristics (eg, title, years funded, principal investigator), PC CDS project phase as per the PC CDS Lifecycle Framework (eg, design and development, implementation and evaluation) [13], type of patient-contributed data collected (eg, PGHD, PROs), description of data collected (eg, step count data from a smartwatch), population addressed with the PC CDS intervention, type of technology used, list of measures used, and data collection methods. The abstraction form can be found in Multimedia Appendix 1.

To identify and extract the list of measures from included studies, the research team used ChatGPT-3.5 to conduct an initial review of each study. A large language model was used to support the initial extraction of performance measures because identifying and cataloging measures required detailed review of a large body of literature (40 studies), including studies in which measures were not consistently labeled or explicitly described. The tool was used as an aid to improve the efficiency and consistency of the extraction process and to facilitate the identification of candidate measures for reviewer assessment, but it was not used as a substitute for human review. Between October and November 2023, research team members developed study-specific prompts that were iteratively refined, as needed, to improve completeness and specificity of extracted information, particularly for qualitative studies in which measures were not explicitly defined (see Multimedia Appendix 2 for the list of baseline prompts). All artificial intelligence (AI)–generated extractions underwent independent review by a research team member (CZ or NG), who compared the output against the full-text paper and corrected or supplemented the extracted information, as needed. Particular attention was paid to verifying accuracy and describing measures at the most specific level possible, including the names of instruments, scales, and PRO questionnaires, when reported. Following quality assurance review, the verified measures were incorporated into the Microsoft Excel abstraction form.

### Key Informant Discussions

We purposively selected 10 projects for key informant interviews with the project principal investigators to gather their perspectives on PC CDS performance measures and measurement-related gaps and challenges. In selecting projects, we aimed to capture diversity in the type of PC CDS technology and patient-contributed data, performance measures evaluated, use case, and phase of the project (eg, design, development, implementation). We also considered recommendations from the AHRQ regarding projects with rich implementation experience that could provide particularly informative perspectives. Of the 10 invited principal investigators, 9 agreed to participate and completed an interview. The high participation rate may have been influenced by knowledge that this study was funded by the AHRQ. To mitigate response bias, interviewers emphasized that participation is voluntary and that responses would be reported at an aggregate level and would not be attributed to individual participants.

We conducted 60-minute individual interviews virtually on Zoom. Interviewers used semistructured discussion guides tailored to each project (see Multimedia Appendix 3). This ensured consistency in data collection, while allowing flexibility to explore unique project contexts and emergent themes. Discussions focused on (1) experiences selecting and using measures to assess PC CDS performance, (2) perspectives on gaps and challenges in PC CDS performance measurement, and (3) perspectives on opportunities to improve PC CDS performance measurement. A senior team member (author PD or DFS) led the discussions, and an analyst recorded transcript-style notes and key takeaways. All interviews were audio-recorded, and all notes were reviewed by four members of the research team (CZ, NG, PD, and DFS) to ensure agreement on key takeaways.

### Technical Expert Panel Consultation

We engaged a TEP convened by the AHRQ-funded Clinical Decision Support Innovation Collaborative (CDSiC), a multiyear project focused on PC CDS, to validate findings and refine interpretations. The TEP included seven experts in CDS, including informaticians, developers, and patient advocates. Over three virtual meetings, we presented interim results and received feedback on (1) comprehensiveness of the identified measures, including notable gaps; (2) relevance of measurement challenges and opportunities raised by key informants; and (3) potential strategies for advancing the field. All meetings were audio recorded (guided discussion questions can be found in Multimedia Appendix 4). An analyst documented detailed meeting minutes, which were refined using the audio recordings and reviewed by senior research team members (PD and DFS) for accuracy. TEP feedback was used to refine findings and inform development of the action plan for PC CDS performance measurement.

### Analysis and Synthesis

We used a sequential synthesis design in which each data collection phase informed subsequent phases of the study to refine our analysis. Specifically, findings from the document review were used to develop and refine the key informant interview guide, enabling us to explore project-specific factors. We then validated and refined our integrated findings with TEP members to support triangulation.

For the document review, two researchers (CZ and NG) independently mapped each measure to the subdomains of an existing PC CDS performance measurement framework and identified measures that did not fit well into the framework [14]. The PC CDS performance measurement framework is informed by constructs from previously established health IT and CDS evaluation frameworks and provides domains and subdomains for measurement to understand the performance and impact of PC CDS technology (Table 1). This framework was selected because it is specifically designed for PC CDS technologies, which require consideration of the full PC CDS lifecycle, patients’ needs and preferences, and outcomes at multiple levels (individual, population, and health system). Several members of the study team (PD, DFS, CZ, and NG) contributed to the framework’s development, and additional testing and independent external validation are needed to further evaluate and refine the framework.

Any discrepancies were resolved through discussion with a third researcher (PD) until a consensus was reached. We used descriptive statistics (eg, frequencies and counts) to summarize measure types across projects to support identification of common areas of measurement and measurement gaps. Additionally, we reviewed each paper’s discussion and limitations sections to identify measurement-related challenges. Using the web-based brainstorming platform Lucidchart [15], which enables real-time collaboration with visual diagramming, two research team members (CZ and NG) abstracted each challenge onto a virtual sticky note and grouped them into broader themes, which were reviewed and refined with a senior researcher (PD).

For the key informant interviews and TEP meetings, we conducted rapid thematic analysis of transcripts and TEP meeting minutes. The lead notetaker reviewed the transcripts and meeting minutes and prepared structured summaries of each interview and TEP meeting that captured higher level themes related to measurement gaps, challenges, and recommendations. After all interview and TEP meeting-specific themes were finalized, two research team members (CZ and NG) grouped them into broader cross-cutting themes. We conducted regular debriefings with the broader study team, including researchers not involved in data collection, to refine emerging themes and reconcile discrepancies through discussion to reach consensus. We conducted a qualitative thematic synthesis to integrate findings across the document review, interviews, and TEP meetings. Themes from the interviews and TEP meetings were compared with those from the document review to validate interpretations, identify gaps and challenges, and enhance the preliminary synthesis. TEP themes also informed the action plan for PC CDS performance measurement.

**Table 1:** Domains and subdomains of the PC CDS^a^ performance measurement framework.

Measurement domain	Measurement subdomains
Safe: Avoiding harm to patients or users resulting from the system itself or the recommendations it provides	Error quantification System quality (eg, free from defects) Completeness
Timely: Reducing waits and sometimes harmful delays (eg, due to technology malfunction) for both those who receive and those who provide care	Availability Computer processing time Up-to-date information provided Information provided when user makes the decision Care timeliness
Effective: Providing functional, accessible systems and services based on scientific knowledge to all who could benefit and refraining from providing systems and services to those not likely to benefit (ie, avoiding underuse and misuse, respectively)	User satisfaction Acceptability Patient health outcomes Clinician performance Transparency Usability
Efficient: Minimizing wasted or unnecessary effort by ensuring the system integrates smoothly into clinician and patient workflows, reduces user burden, and supports the intended user	Relevance and appropriateness Interoperability Cost Reuse and scalability Use of services Cognitive workload
Equitable: Providing interventions, advice, and care that account for social determinants of health and do not vary in quality because of personal characteristics	Social context Economic context Health literacy Digital health literacy Physical infrastructure Health care context
Patient centered: Providing interventions, advice, and care that are respectful of and responsive to individual patient preferences, needs, and values and ensuring patient values guide all clinical decisions	Patient activation Patient engagement Patient satisfaction Patient decision-making Patient-relevant outcomes Decisional quality Patient knowledge acquisition

^a^PC CDS: patient-centered clinical decision support.

### Ethical Considerations

The NORC at the University of Chicago Institutional Review Board (FWA00000142) reviewed all methods and deemed this work not human subjects research. Key informant interview participants were informed about the purpose of the study, the voluntary nature of participation, and the planned use of study data. Verbal informed consent was obtained from all participants prior to conducting interviews. Interview and TEP meeting recordings and transcripts were stored in a secure NORC environment with access restricted to authorized research team members. To protect confidentiality, participants were anonymized and findings synthesized at the aggregate level. No compensation was provided to participants for their involvement in the study.

## Results

### Project Characteristics

Figure 1 shows the flowchart for identifying projects via the AHRQ’s Digital Healthcare Research Program. Details of the 20 projects [16-35] included in the review can be found in Multimedia Appendix 5. The projects involved PC CDS technologies in stages of (1) design, (2) development, (3) implementation, and (4) evaluation (Figure 2). The design and development phases involve identification of user needs and requirements, preliminary designs, rapid prototyping with user input, user testing and process, and technology changes prior to implementation [36,37]. The implementation phase involves the deployment of the PC CDS into clinical workflows [36-38] or patients’ daily activities, and the evaluation phase is the process of measuring whether the tool achieves its defined objectives [36,38,39]. Most projects (n=12, 60%) covered all phases of the PC CDS lifecycle, while some focused only on implementation and evaluation (n=7, 35%) or design and development (n=1, 5%).

**Figure 1:**
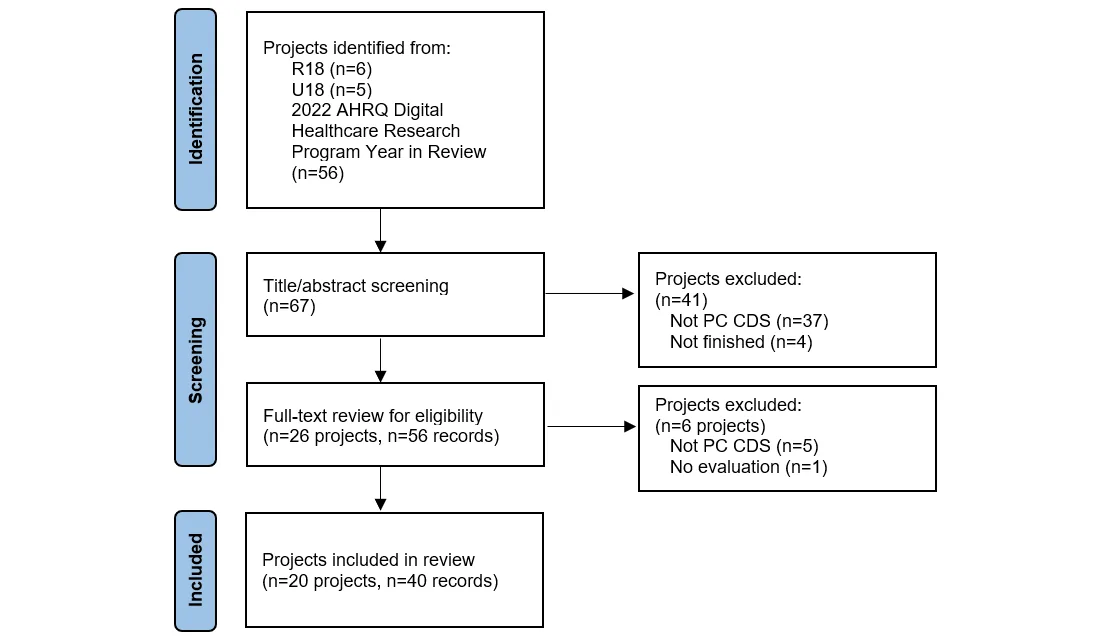
Identification of projects via the AHRQ's Digital Healthcare Research Program. AHRQ: Agency for Healthcare Research and Quality; PC CDS: patient-centered clinical decision support.

**Figure 2:**
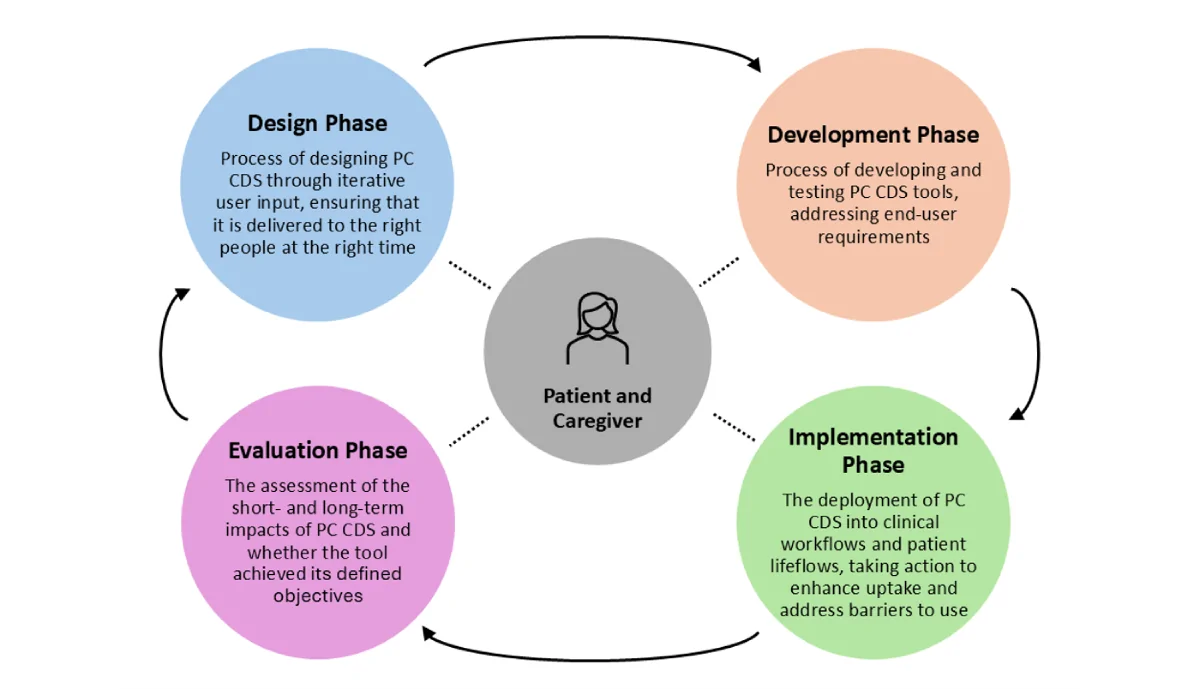
PC CDS lifecycle phases covered in reviewed projects. PC CDS: patient-centered clinical decision support.

The PC CDS technologies addressed various medical conditions and/or HRSN, with the most common being chronic conditions (n=11, 55%), such as arthritis, asthma, cancer, diabetes, or HIV. Other focus areas included health behavior/education, such as diet, medication adherence, physical activity, or preconception care (n=4, 20%); mental health or neurologic conditions, such as attention-deficit hyperactivity disorder, depression, or dementia (n=3, 15%); social needs, such as changes in life circumstances and behaviors or intimate partner violence (n=2, 10%); and acute conditions, such as dental pain or gastrointestinal bleeding (n=2, 10%).

The projects used different types of patient-contributed data to inform PC CDS interventions, with the most common being PROs for specific conditions, such as depression, quality of life, and pain (n=13, 65%). Other types included PGHD collected by devices, such as blood pressure and step count (n=4, 20%); HRSN, such as job loss (n=3, 15%); and patient preferences, such as treatment options (n=1, 5%). In terms of the technology used, the projects frequently leveraged mobile apps/smartwatches (n=9, 45%) and electronic health records (EHRs; n=7, 35%), while a few used machine learning (ML) or AI (n=3, 15%), web-based tools (n=3, 15%), or text messaging (n=2, 10%). Patients were the most common end users of the technologies (n=18, 90%), although many were both patient- and clinician-facing technologies (n=13, 65%), with 2 (10%) being developed for caregivers.

When the type of patient-contributed data and type of technology were examined together, we found that projects collecting and using PROs leveraged all four types of technology (mobile apps/smartwatches, EHRs, AI/ML, and web-based tools), as shown in Figure 3. All PGHD were collected and shared using mobile apps/smartwatches or text messaging directed to the patient. All projects using HRSN data did so by using EHRs for collection and display. Only 1 (5%) project collected patient preference data using the EHR.

**Figure 3:**
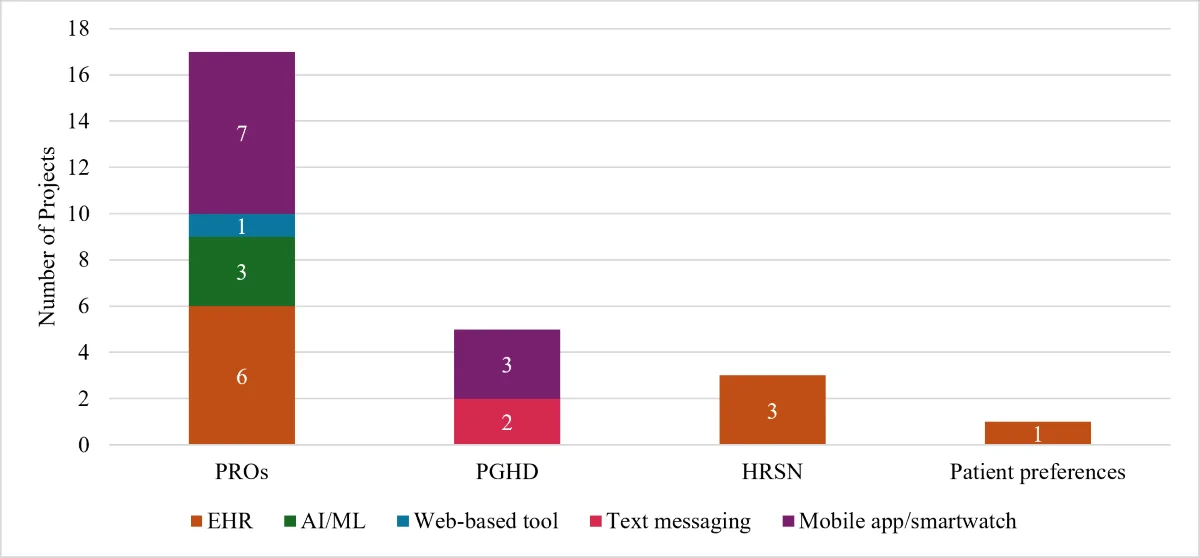
Type of technology and patient-contributed data used in projects. AI: artificial intelligence; EHR: electronic health record; HRSN: health-related social needs; ML: machine learning; PGHD: patient-generated health data; PRO: patient-reported outcome.

### Key Findings from the Document Review

Here, we present common measurement areas in PC CDS projects, identified through our document review, across both the design and development and the implementation and evaluation phases. Measurement in the design and development phases focused on assessing end-user needs and preferences. Measurement in the implementation and evaluation phases extended beyond usability to assess impact and effectiveness.

#### Common Measures in PC CDS Design and Development From Selected Studies

Measurement in the design and development phases of PC CDS technologies prioritized capturing the needs, experiences, preferences, and priorities of key end users (ie, patients, caregivers, and/or clinicians) to support the development or refinement of prototypes and ensure alignment with patient needs. Projects commonly used qualitative approaches, such as key informant interviews, focus groups, think-aloud sessions, and participatory design sessions, to assess end-user needs. Only a few projects used surveys or Likert scales to quantitatively assess user needs and perspectives.

- Usability: Projects most frequently measured the usability of PC CDS (n=10, 50%), or the degree to which the technology enabled users to carry out their tasks safely, effectively, or efficiently [40]. This includes usage, or the degree to which the technology is or will be used [40], and user satisfaction, or the degree to which patients, caregivers, or clinicians are satisfied with their experience in using the technology and with the technology’s potential impact. Patients provided feedback on sample text messages, communication preferences, the ease of use of graphical displays, and suggestions for improvement. For example, children with attention-deficit hyperactivity disorder were directly engaged in sharing feedback and sketching designs for a wearable self-management app to assess and improve usability [41]. Clinicians provided feedback on the time needed to use the technology, technological challenges experienced, anticipated clinical utility, and impact on clinical workflows.
- Acceptability: For acceptability (n=5, 25%) projects assessed the degree to which the clinician or patient perceived the technology as appropriate, adequate, and relevant [14,38,42]. One project designing a PRO collection app gathered feedback from younger patients with higher education and technology use as well as from older patients with lower education and technology use to understand concerns about data privacy and transparency. The project also assessed clinicians’ perspectives on their interest in using PRO physical function data to support clinical care [43].

#### Common Measures in PC CDS Implementation and Evaluation from Selected Studies

Although usability remained a central focus in all projects, measurement in the implementation and use phases also extended to assess effectiveness and clinical impact. Measures were both qualitative and quantitative.

- Usability: For usability (n=15, 75%), measures of use and ease of use of the technology were the most common. In contrast to the design and development phases, projects in this phase frequently used quantitative approaches through surveys, such as the System Usability Scale [44], and frequency estimates, such as the number of user logins, percentage of users accessing the tool, or time spent using the technology or specific features. Projects also used qualitative approaches to gather user feedback on barriers to and facilitators of technology use and ease of use.
- Patient health outcomes: For patient health outcomes (n=14, 70%), changes in PRO scores were often used to assess the impact of interventions. Several projects administered condition-specific PRO questionnaires, such as the Functional Assessment of Cancer Therapy – General-7 Item Version [45], as well as general behavioral health and quality-of-life questionnaires, such as the Patient Health Questionnaire-4 [46], and various versions of the Patient-Reported Outcomes Measurement Information System [47]. For example, in one project evaluating a mobile app for asthma self-management, researchers administered the Asthma Control Test [48] and the Mini Asthma Quality of Life Questionnaire [49] at multiple time points to assess longitudinal effects. Additionally, projects measured changes in laboratory values for conditions with well-established clinical biomarkers (eg, CD4 cell count, hemoglobin A1C). Projects that focused on collecting HRSN measured outcomes including estimated days of life saved and the frequency of compliance to United States Preventive Services Task Force guidelines (eg, lung cancer screening).
- Patient engagement: Projects measured patient engagement (n=11, 55%) in various ways. Some projects measured engagement with the technology with traditional usage metrics (eg, days of use, time span between use, response rates, or actions taken within the tool). A few projects assessed patients’ engagement through study attrition rates. Several projects assessed changes in patients’ behaviors as a result of their interactions with PC CDS. This included medication adherence, physical activity, communications with their care team, attendance at primary care visits, and the Stage of Change questionnaire.
- Clinician performance: For clinician performance (n=8, 40%), researchers often looked at how clinicians responded to the PC CDS tool in terms of response time and adherence to alerts, follow-up actions, and treatment decisions. These measures were more common in projects involving EHR-based technology with a clinician-facing decision support component.

Table 2 provides more details on the common measures in each phase.

**Table 2:** Common measures based on the PC CDS^a^ phase.

Phase and measure subdomains^b^		Illustrative measures
**Design and development**		
	Usability	Desired features and functionalities [16,43,50] Perceived barriers and concerns related to use [16,43] Perceived utility [17,43,51] Issues using or comprehending information provided by design prototypes [18,43] Overall experience using PC CDS technology [19]
	Acceptability	Receptiveness to PC CDS technology [20] Degree of interest in use of PC CDS technology and PGHD^c^ [43]
**Implementation and evaluation**		
	Usability	Ease of use [50,52,53] PC CDS technology use [20-23,50] Barriers to use and suggestions for improvement [16,20,21] Overall experience using PC CDS technology [16,24,52,54]
	Patient health outcomes	Changes in physical symptoms or lab indicators [16,17,20,21,24,52] Changes in mental health [17,20]
	Patient engagement	Degree of engagement with PC CDS technology [50] Change in patient-provider communication [20] Patient behavior change [23]
	Clinician performance	Alert compliance and follow-up [52] Adherence to recommendations of a PC CDS tool (eg, risk calculator, screening tool) [25] Appropriate treatment decisions made based on patient-reported information (eg, medications prescribed) [26]

^a^PC CDS: patient-centered clinical decision support.

^b^Measure subdomains taken from Dullabh et al [14].

^c^PGHD: patient-generated health data.

### Key Findings from Key Informant Interviews

To explore gaps in PC CDS measurement, we asked key informants to elaborate on those identified in the document review and to provide additional insights based on their research and practice. Here, we describe several key gaps and challenges in measurement across PC CDS phases:

- Few projects conducted ongoing monitoring of the safety and timeliness of PC CDS technology (eg, technical performance, quality of information presented, unintended consequences) postimplementation. Relatively few projects conducted ongoing assessments of the PC CDS technology’s ability to reduce potential harm to patients or avoid delays in care postimplementation [26]. During design and development, a few projects assessed the accuracy and completeness of the information generated by the PC CDS technology by asking patients and experts to rate the quality of messages and by conducting think-aloud sessions with clinicians for a prototype. In addition, informants described how projects routinely test the technology’s performance during design and development, but many studies did not frequently report those results. For the few projects that conducted postimplementation monitoring of PC CDS technology, measures focused on the frequency and timeliness of alerts, the time spent with patients, and patient-reported adverse events or issues with PC CDS.
- Standard usability measurement approaches during design and development are not well suited to populations with limited digital or health literacy. Key informants reported that traditional usability testing methods, such as card sorting or structured forms, were not well suited to individuals with limited digital or health literacy due to the cognitive demand of sorting medical terms and unfamiliarity with smartphone features [55]. In response, their teams used qualitative interviews to capture participants’ experiences as a more accessible approach. Although effective, a few key informants reported this approach may be more time- and resource-intensive for both the researcher and the participant, leading to increased costs and longer design and development phases.
- PC CDS technologies that store patient data on third-party servers present privacy concerns for health systems, limiting measurement of patient engagement. Projects using third-party cloud-based PC CDS platforms encountered health system reluctance to allow storage of protected health information (PHI) outside institutional firewalls. This frequently impeded the collection of individual-level data on use and engagement with technologies, unless projects could implement and receive health system approval of privacy-protecting processes to handle the data (eg, maintaining access logs or records of disclosures). In cases where this was not likely or too time-consuming, researchers instead relied on self-reported or aggregate-level measures of use, limiting the ability to conduct more sophisticated analyses of how individual engagement impacts intervention effectiveness.
- For clinician-facing apps, data are documented in disparate places in EHRs, making it challenging and costly to consistently implement PC CDS and accurately measure clinician use. Relevant clinical data, clinician actions, and documentation essential for PC CDS functionality were dispersed across numerous places in EHRs, requiring customization to integrate these tools effectively. This complicated both the deployment of PC CDS technology and the extraction of meaningful usage data. Key informants described conducting manual chart abstraction, developing custom queries, and performing iterative data pulls, which are complex and costly. In addition, clinician usage data were often captured in EHR audit logs, which tend to be quite voluminous and difficult to parse.
- The limited scale and formative nature of PC CDS projects hinder measurement of long-term impact on patient health outcomes. Many projects focused on near-term or process-oriented outcomes (eg, screening uptake, knowledge change, medication adherence), which were more feasible within the scope of project funding and timelines. Few studies in our sample incorporated control arms or randomized designs, making it difficult to draw causal conclusions. Key informants emphasized the importance of assessing longer-term clinical outcomes to determine the true impact of PC CDS. Although randomized controlled trials (RCTs) remain the gold standard for establishing causation, they are difficult and costly to conduct, often requiring extended follow-up periods, and large sample sizes.
- Cost measures can be too complex and resource-intensive to operationalize within project timelines and budgets. In our review, we only identified two measures related to cost: (1) direct medical costs attributed to misuse, overuse, and underuse of medical services [27], and (2) patient-reported financial burden from health care usage, as measured by the Comprehensive Score for Financial Toxicity–Functional Assessment of Chronic Illness Therapy (COST-FACIT) questionnaire [56]. Key informants attributed this to the methodological complexity and resource demands of measuring cost. Informants described that measuring costs during the design and development phases was more feasible as teams could clearly account for developer costs, but operational costs, which vary by health system and EHR system, were much harder to account for. Measuring patient-level financial impacts (eg, transportation or data charges) and long-term cost savings from improved health outcomes and/or quality of life were often not feasible, given the short duration and resources of most projects. Some informants reported undertaking more advanced analyses in subsequent work, such as cost-effectiveness studies or microcost tracking using sensor technology, but these efforts typically required additional funding and expertise. Key informants also noted that cost is often excluded simply because many funding mechanisms do not prioritize or require economic evaluation.
- Lack of standardized measures and benchmarks prevent meaningful, generalizable measurement of patient engagement with PC CDS technology. Projects varied widely in how they defined and measured patient engagement with PC CDS, limiting comparability and generalizability. The project documents, key informants, and TEP members all described a need for standardized measures of both engagement processes (eg, technology use) and outcomes (eg, behavior change and engagement with care), as well as clear benchmarks for what should be considered a high level of engagement. Key informants raised several challenges related to patient engagement measurement. They noted that PC CDS tools disproportionately engage patients already active in their care, making it difficult to understand or reach less engaged populations. Informants also discussed fragmented data sources, as patients often receive care across multiple systems, and technical limitations of some tools (eg, low-frequency data capture in smartphone-based pedometers). Self-reported measures of engagement introduced uncertainty due to recall and selection bias.

### Validation and Refinement from the Technical Expert Panel

TEP members provided guidance and feedback on preliminary findings during the beginning, middle, and end of the study. Overall, the panel responded positively to the findings and action plan, affirming their relevance, while pointing to key opportunities to strengthen measurement approaches, standardization, and evidence generation in the PC CDS field.

Engagement emerged as a central theme, with members underscoring both its importance and complexity. They observed that patient engagement varies significantly depending on clinical context, with patients experiencing acute or high-salience conditions (eg, pregnancy or cancer) often demonstrating higher engagement than those managing chronic conditions. Members also discussed the need for better standardization of engagement measures and distinguishing between process measures (eg, onboarding steps, such as downloading an app or logging in) and outcome measures (eg, whether patients take an intended action). However, attributing outcomes directly to PC CDS remained challenging due to confounding factors, reinforcing the need for rigorous study designs (eg, A/B testing, control groups) and improved statistical approaches. They also highlighted the importance of comparing engagement results to baselines or benchmarks and accounting for organizational scale. For example, what may appear to be modest engagement rates could still translate into meaningful improvements in reach and cost savings in large health systems.

Members also provided insights into gaps in current PC CDS measurement. Similar to key informants, TEP members suggested that limited attention to domains such as cost and technical performance may reflect the early-stage nature of many PC CDS projects, which are often focused on feasibility rather than long-term performance or scalability. At the same time, members found it notable that technical performance is not more consistently measured, given its importance for patient trust and system usability.

## Discussion

### Principal Findings

Our review sought to identify measures currently used to evaluate PC CDS technologies in federally funded digital health projects and to characterize gaps in existing measurement approaches as they are increasingly integrated into clinical care. We found that usability, acceptability, patient health outcomes, patient engagement, and clinician performance were the most commonly measured domains in the selected PC CDS studies, while relatively little attention was given to technology safety, timeliness, and cost. This is notable because these domains may substantially influence the sustainability, scalability, and real-world value of PC CDS technologies [57,58]. The absence of these measures suggests that current measurement approaches may provide an incomplete picture of PC CDS performance, particularly as tools move from research settings into routine clinical practice.

We also identified persistent challenges in measuring some of the commonly assessed domains that underscore the unique complexities of evaluating PC CDS technologies that interact directly with patients. Projects included in this review found that existing usability instruments are often not well suited for patient populations with limited digital health literacy, a concern that has been raised in digital health research more broadly [59] but may be particularly consequential for PC CDS because these technologies depend on patients’ ability to interpret and act on health information outside of traditional clinical encounters. Additionally, although the prior literature has highlighted the importance of patient engagement for the success of digital health interventions [60], our review identified that measuring patient engagement for PC CDS specifically is complicated by the use of third-party applications that support these technologies and health system privacy and security concerns that restrict their collection and use of data.

Other challenges identified in this review mirror longstanding issues in health information technology evaluation. Assessment of patient health outcomes is frequently limited by the short duration and scale of many studies, while measuring clinician use of technologies remains difficult because relevant data are often embedded within complex EHR systems. Although these barriers are not unique to PC CDS, our review demonstrates how they continue to inhibit measurement and therefore our understanding of how technologies can improve care.

Although the projects in this review may not be applicable across all PC CDS contexts, collectively, these findings indicate six important next steps for future development of PC CDS performance measurement as the field continues to mature:

- Encourage the measurement and reporting of technical performance postimplementation. Technical performance of PC CDS technologies was more often measured by developers during testing activities during the technology’s design and development, while only a few studies conducted ongoing measurement postimplementation. Implementers could benefit from understanding how the technology performs once in production, particularly when delivered to patients via apps, patient portals, or text messaging. Monitoring and measurement after implementation can reveal important insights into how the technology’s performance impacts patient engagement and safety in a real-world setting, for example, with varying levels of internet speed and availability or when underlying EHR configurations change [61]. Researchers have recommended developing continuous automated monitoring dashboards to identify unusual patterns before they are noticed by end users, such as when CDS firing suddenly stops, to maintain trust in the CDS [61,62]. For patient-facing tools, failures may go unnoticed by clinicians but still erode patient trust and participation. Although our review only included a few AI-/ML-supported technologies, ongoing monitoring will be especially critical with the growth of these tools, which can experience algorithmic drift and degraded performance over time, both of which are difficult to identify [63]. Further, monitoring the performance of a technology once deployed would enable health systems to more efficiently choose the correct tools and interventions for their population or setting.
- Conduct evaluations of real-world projects in low-resource health care settings to assess what is needed to effectively implement PC CDS technology. Many of the selected PC CDS studies are still formative in nature and focus on near-term outcomes in small samples or single-site settings. We found few RCTs or long-term evaluations across different populations and settings. More real-world implementation projects are needed in rural, public, and safety net settings to understand which technologies are best suited to address different health care conditions and settings, what is needed for a health care system to adopt PC CDS technologies, and how technologies perform outside of a controlled research setting. Conducting evaluations in a wider range of settings will also advance our understanding of the types of patient users and what measures and methods are best suited to evaluate their perspectives of PC CDS technologies.
- Develop practical approaches to cost assessments to support wider use and adoption of PC CDS interventions. Cost analyses were rare and primarily focused on the effects of PC CDS on health care use costs rather than holistic economic evaluations of implementation. Given the variability in funding resources and timelines mentioned by key informants, identifying efficient, easy-to-use methods for quantifying the cost of developing and implementing PC CDS technology will be critical to assess value without requiring intensive methods. These methods should account for the different phases of the PC CDS lifecycle, from development through dissemination, and be flexible enough to support decision-making across organizations of different sizes and resources. This could include tools such as cost calculators, models, frameworks, or priority measures for assessing value [57]. A systematic review identified value areas provided by CDS systems, including quality assurance, clinical benefits, user satisfaction, guideline adherence, and more, yet the need for an organized framework to further identify all CDS value areas was noted [64]. In addition to developing practical tools, other strategies may help address resource and time barriers, such as bundling multiple PC CDS tools aimed at a shared quality improvement target to assess collective value at a broader scale or conducting small-scale pilots (eg, mini RCTs) to assess value prior to scaling [57]
- Consider approaches to managing health systems’ privacy and security concerns with storing individual data on external apps and servers. Usability and patient engagement were commonly measured in the selected PC CDS studies but may only provide a partial picture of the technology’s use and effectiveness, given concerns related to storing data on third-party patient-facing applications. For these areas to be truly understood, more work is needed to safely capture, store, and use PHI available from patient-facing apps. Currently, health system PC CDS projects collecting PHI through patient-facing apps (eg, individual use data) and storing those data on external servers often use service-level agreements that require maintaining access logs and records of disclosure to comply with federal Health Insurance Portability and Accountability Act regulations. App developers can use the Federal Trade Commission’s Mobile Health App Interactive Tool to explore which federal laws and regulations may apply to their app [65]. Emerging standards, such as SMART Health Cards and SMART Health Links [66], also offer privacy-preserving methods for patients to share PHI directly with health care systems, although they are not designed for longitudinal or continuous collection of app use and other PHI and so may be useful in more select contexts. Future work should explore how these consent-driven data-sharing mechanisms can be adapted to support PC CDS performance measurement.
- Refine patient engagement measures and develop standardized benchmarks to assess who is engaging with PC CDS technologies, how well they are engaging, and how engagement impacts outcomes. Patient engagement measurement was highly variable across studies and focused more on processes (ie, use of technology) rather than meaningfulness. Despite the large amount of literature on patient engagement, there is a need for more measures to assess how PC CDS technologies support users’ actions, empowerment, and engagement in care. Health systems would benefit from standardized benchmarks regarding what is considered high or low engagement with a PC CDS technology to know how their technology performs in this area. They would also like to understand the full process of how participants engage with PC CDS technology and how they overcome hurdles along the way (eg, logging in, navigation, inputting data) through more qualitative research or correlations with meaningful outcomes. For instance, if a patient uses a PC CDS tool to report symptoms and receives tailored guidance to seek care, tracking whether that engagement is associated with a future clinical encounter could help determine whether the tool is influencing care-seeking behavior. There are also several instruments (eg, Patient Engagement in Research Scale [67], Research Engagement Survey Tool [68]) that measure patient/partner engagement throughout a research study that could be adapted when conducting PCOR for PC CDS. Ultimately, researchers would like a better understanding of how engagement with PC CDS technology impacts health outcomes both in the short and the long term [69].
- Adapt assessment tools and methods for populations with limited English proficiency and digital and/or health literacy. Traditional usability measurement tools may not be effective for all populations, particularly those with limited digital literacy, health literacy, or digital health literacy levels, so researchers tend to rely on qualitative methods to solicit feedback from these populations. Additionally, some traditional user-centered design approaches (eg, card sorting activities, open brainstorming) may be cognitively demanding for those with communication barriers [55]. Although there have been recent advancements in frameworks and toolkits to guide researchers in user-centered design for different contexts [70,71], having access to more survey tools, instruments, and measurement methods tailored for individuals with limited English proficiency, digital literacy, and/or health literacy would likely increase engagement of these populations and produce more meaningful findings that advance the field of PC CDS as a whole [72]. For example, the System Usability Scale, a popular usability survey, could be adapted with appropriate testing to remove or modify questions that are not contextually appropriate or that use unfamiliar terminology [73].

### Limitations

This study has some limitations. The document review was restricted to a single funding program, the AHRQ’s Digital Healthcare Research Program, that aims to advance digital health care technologies that address the evolving needs of patients, clinicians, and health systems. This limits the generalizability of findings to other funding mechanisms or research settings. Additionally, the key informant interviews were conducted with a purposively selected subset of the included projects to capture a range of PC CDS technologies, patient-contributed data, and evaluation experiences. As such, the interview findings may not reflect the perspectives of all projects within the portfolio or the broader PC CDS field. Third, participation in interviews was voluntary, introducing the potential for selection bias in interview participation, as principal investigators who agreed to be interviewed may represent a particular perspective on their own work. Finally, interview responses may have been impacted by self-report biases, as investigators may have been reluctant to acknowledge challenges or shortcomings within their own projects and may attribute limitations to external factors instead.

### Conclusion

This study identified PC CDS performance measurement areas that are well defined and represented in 20 real-world PC CDS projects, as well as gaps that limit our understanding of how these technologies perform in practice. Although we only reviewed a small subset of PC CDS projects in the formative research stage, the findings suggest that there is not yet an established, comprehensive approach to evaluating PC CDS technologies across different patient populations, settings, and stages of implementation. To advance the development and use of PC CDS, future efforts should (1) measure technical performance after implementation to understand how tools function in real-world use; (2) evaluate interventions in low-resource health care settings to account for varied infrastructure and capacity; (3) conduct more cost assessments to inform feasibility and scalability; (4) develop practical approaches to managing health systems’ privacy and security concerns with storing individual data on external apps and servers; (5) refine and develop standardized patient engagement measures to assess who is engaging with PC CDS technologies, how well they are engaging, and how engagement impacts outcomes; and (6) adapt measurement tools to populations with limited English proficiency or digital and/or health literacy. Addressing these areas can strengthen the evidence base for PC CDS and support development of technologies that promote patient-centered care.

## Appendix

Multimedia Appendix 1 Data abstraction tool used during project document review.

Multimedia Appendix 2 ChatGPT prompts for measurement abstraction.

Multimedia Appendix 3 Key informant interview discussion guide and protocol.

Multimedia Appendix 4 Technical expert panel meeting discussion questions.

Multimedia Appendix 5 Study characteristics.

## Abbreviations

AHRQ: Agency for Healthcare Research and Quality
AI: artificial intelligence
CDS: clinical decision support
EHR: electronic health record
GAI: generative artificial intelligence
HRSN: health-related social needs
ML: machine learning
PC CDS: patient-centered clinical decision support
PCOR: patient-centered outcomes research
PGHD: patient-generated health data
PHI: protected health information
PRO: patient-reported outcome
RCT: randomized controlled trial
TEP: technical expert panel

## Data Availability

The datasets generated and analyzed during this study are not publicly available due to possible reidentification.

## Use of Generative Artificial Intelligence

The authors declare the use of generative artificial intelligence (GAI) in the research. According to the GAIDeT taxonomy (2025), the following task was delegated to GAI tools under full human supervision: data collection (GAI tool used: ChatGPT 3.5).

Responsibility for the final manuscript lies entirely with the authors. GAI tools are not listed as authors and do not bear responsibility for the final outcomes.

The authors used ChatGPT 3.5 to extract performance measures from each included study. Research team members (authors CZ and NG) then reviewed the extracted measures against the full text of each study for accuracy and completeness. After this review, they added them to the data extraction spreadsheet.

## References

[1] About CDSiC. Agency for Healthcare Research and Quality.

[2] Dullabh P, Sandberg SF, Heaney-Huls K, Hovey LS, Lobach DF, Boxwala A, Desai PJ, Berliner E, Dymek C, Harrison MI, Swiger J, Sittig DF (2022). Challenges and opportunities for advancing patient-centered clinical decision support: findings from a horizon scan. J Am Med Inform Assoc.

[3] Clinical decision support. Agency for Healthcare Research and Quality.

[4] Clinical decision support. Assistant Secretary for Technology Policy/Office of the National Coordinator for Health Information Technology.

[5] CDS- clinical decision support. Centers for Medicare & Medicaid Services.

[6] (2025). Request for information; health technology ecosystem. Federal Register.

[7] Dullabh P, Heaney-Huls K, Lobach DF, Hovey LS, Sandberg SF, Desai PJ, Lomotan E, Swiger J, Harrison MI, Dymek C, Sittig DF, Boxwala A (2022). The technical landscape for patient-centered CDS: progress, gaps, and challenges. J Am Med Inform Assoc.

[8] Dullabh P, Zott C, Gauthreaux N, Aronoff A, Sittig DF (2025). Chapter 5. Measurement concepts across the PC CDS lifecycle. Innovation Center: Framework and Inventory of Patient Engagement Measures for PC CDS.

[9] Denzin NK, Lincoln YS (2011). The SAGE Handbook of Qualitative Research. 4th ed.

[10] Thomas J, Harden A (2008). Methods for the thematic synthesis of qualitative research in systematic reviews. BMC Med Res Methodol.

[11] Digital healthcare research. Agency for Healthcare Research and Quality.

[12] Hacker K, Houry D (2022). Social needs and social determinants: the role of the Centers for Disease Control and Prevention and public health. Public Health Rep.

[13] Sittig DF, Boxwala A, Wright A, Zott C, Desai P, Dhopeshwarkar R, Swiger J, Lomotan EA, Dobes A, Dullabh P (2023). A lifecycle framework illustrates eight stages necessary for realizing the benefits of patient-centered clinical decision support. J Am Med Inform Assoc.

[14] Dullabh P, Zott C, Gauthreaux N, Swiger J, Lomotan E, Sittig DF (2025). New performance measurement framework for realizing patient-centered clinical decision support: qualitative development study. J Med Internet Res.

[15] Home page. Lucidchart.

[16] Lakes KD, Hayes GR, Schuck SEB, Cibrian F, Nelson M (2022). The CoolCraig app: promoting health by improving self-regulation in adolescents with ADHD. Agency for Healthcare Research and Quality.

[17] Clinical decision support for collaborative diet goal setting in primary care. Agency for Healthcare Research and Quality.

[18] Gold H, Bertini E (2021). Development and evaluation of patient-reported outcome score visualization to improve their utilization (PROVIZ). Agency for Healthcare Research and Quality.

[19] Snyder ME, Chewning B, Kreling D, Perkins SM, Knox LM, Adeoye-Olatunde OA, Jaynes HA, Schommer JC, Murawski MM, Sangasubana N, Hillman LA, Curran GM (2021). An evaluation of the spread and scale of PatientToc™ from primary care to community pharmacy practice for the collection of patient-reported outcomes: a study protocol. Res Social Adm Pharm.

[20] Wells BJ, Sandberg JC, Miller Jr DP, McClain DA (2022). A direct-to-patient alert for glycated hemoglobin screening using prediction modeling and mobile health (mHealth). Agency for Healthcare Research and Quality.

[21] Jariwala SP, Feldman J, Reznik M, Rosenstreich D, Mirhaji P, Mowrey W, Zheng K, Sindhu S (2022). Adapting, scaling, and spreading an algorithmic asthma mobile intervention to promote patient-reported outcomes within primary care settings. Agency for Healthcare Research and Quality.

[22] Lin E, Uhler LM, Finley EP, Jayakumar P, Rathouz PJ, Bozic KJ, Tsevat J (2022). Incorporating patient-reported outcomes into shared decision-making in the management of patients with osteoarthritis of the knee: a hybrid effectiveness-implementation study protocol. BMJ Open.

[23] Garcia SF, Penedo FJ, Bass M, Cella D, Kircher S, Smith JD, Webster KA (2022). Expansion, implementation, and evaluation of electronic health record-integrated patient-reported symptom screening in a comprehensive cancer center. Agency for Healthcare Research and Quality.

[24] Aguilera A, Lyles C, Garcia F (2022). Diabetes and Depression Text Messaging Intervention (DIAMANTE). Agency for Healthcare Research and Quality.

[25] Kawamoto K, Caverly T, Del Fiol G, Fagerlin A, Flynn M, Hess R, Kukhareva P, Martin D, Ranade P, Reddy C, Weir C, Zhang Y (2022). Scalable decision support and shared decision making for lung cancer screening. Agency for Healthcare Research and Quality.

[26] Kalenderian E, Walji M, White JM, Yansane A-I, Skourtes N, Mullins J, Brandon R, Holmes D (2022). Optimizing acute post-operative dental pain management using new health information technology. Agency for Healthcare Research and Quality.

[27] Weiner SJ, Schwartz A, Weaver F, Galanter W, Olender S, Kochendorfer K, Binns-Calvey A, Saini R, Iqbal S, Diaz M, Michelfelder A, Varkey A (2022). Integrating contextual factors into clinical decision support to reduce contextual error, improve outcomes, and reduce misuse, overuse, and underuse of outpatient primary care. Agency for Healthcare Research and Quality.

[28] WISE app trial: improving health outcomes in people living with HIV. Agency for Healthcare Research and Quality.

[29] Advancing the collection and use of patient-reported outcomes through health information technology. Agency for Healthcare Research and Quality.

[30] Examining the clinical workflow and outcomes of integrating health information technology to educate and support dementia caregivers. Agency for Healthcare Research and Quality.

[31] EHR-based screening and intervention for intimate partner violence. Agency for Healthcare Research and Quality.

[32] Integrating patient-reported outcomes into routine primary care: monitoring asthma between visits. Agency for Healthcare Research and Quality.

[33] Rheumatology Informatics System for Effectiveness Patient-Reported Outcome (RISE PRO) dissemination project. Agency for Healthcare Research and Quality.

[34] Jack BW, Paasche-Orlow MK, Doros G Implementation and dissemination of 'Gabby,' a health information technology system for young women, into community-based clinical sites. Agency for Healthcare Research and Quality.

[35] Enabling shared decision making to reduce harm from drug interactions: an end-to-end approach. Agency for Healthcare Research and Quality.

[36] Kukhareva PV, Weir C, Del Fiol G, Aarons GA, Taft TY, Schlechter CR, Reese TJ, Curran RL, Nanjo C, Borbolla D, Staes CJ, Morgan KL, Kramer HS, Stipelman CH, Shakib JH, Flynn MC, Kawamoto K (2022). Evaluation in Life Cycle of Information Technology (ELICIT) framework: supporting the innovation life cycle from business case assessment to summative evaluation. J Biomed Inform.

[37] Moullin JC, Dickson KS, Stadnick NA, Rabin B, Aarons GA (2019). Systematic review of the Exploration, Preparation, Implementation, Sustainment (EPIS) framework. Implement Sci.

[38] Van de Velde S, Kunnamo I, Roshanov P, Kortteisto T, Aertgeerts B, Vandvik PO, Flottorp S, GUIDES expert panel (2018). The GUIDES checklist: development of a tool to improve the successful use of guideline-based computerised clinical decision support. Implement Sci.

[39] Ammenwerth E, Brender J, Nykänen P, Prokosch H, Rigby M, Talmon J, HIS-EVAL Workshop Participants (2004). Visions and strategies to improve evaluation of health information systems. Reflections and lessons based on the HIS-EVAL workshop in Innsbruck. Int J Med Inform.

[40] Lau FY, Kuziemsky C (2016). Handbook of eHealth Evaluation: An Evidence-Based Approach.

[41] Cibrian FL, Lakes K, Schuck S, Tavakoulnia A, Guzman K, Hayes G (2020). Supporting self-regulation of children with ADHD using wearables: tensions and design challenges. https://doi.org/10.1145/3313831.3376837.

[42] Xavier PB, Silva ÍS, Dantas THDM, Lopes RH, de Araújo AJ, de Figueirêdo RC, da Costa Uchôa SA (2024). Patient satisfaction and digital health in primary health care: a scoping review protocol. Front Public Health.

[43] Wesley DB, Schubel L, Hsiao C, Burn S, Howe J, Kellogg K, Lincoln A, Kim B, Ratwani R (2019). A socio-technical systems approach to the use of health IT for patient reported outcomes: patient and healthcare provider perspectives. J Biomed Inform.

[44] Brooke J, Jordan PW, Thomas B, Weerdmeester BA, McClelland IL (1996). SUS: a quick and dirty usability scale. Usability Evaluation in Industry.

[45] FACT-G7: functional assessment of cancer therapy – general – 7 item version. FACIT.org.

[46] Kroenke K, Spitzer RL, Williams JB, Löwe B (2009). An ultra-brief screening scale for anxiety and depression: the PHQ-4. Psychosomatics.

[47] PROMIS. HealthMeasures.

[48] Home. Asthma Control Test.

[49] Juniper E, Guyatt G, Cox F, Ferrie P, King D (1999). Development and validation of the Mini Asthma Quality of Life Questionnaire. Eur Respir J.

[50] Rudin R, Perez S, Rodriguez J, Sousa J, Plombon S, Arcia A, Foer D, Bates DW, Dalal AK (2021). User-centered design of a scalable, electronic health record-integrated remote symptom monitoring intervention for patients with asthma and providers in primary care. J Am Med Inform Assoc.

[51] Wood OR, Schnall R, Kay ES, Jia H, Abua JA, Nichols TK, Olender SA, Mugavero MJ, Batey DS (2023). A community health worker and mobile health app intervention to improve adherence to HIV medication among persons with HIV: the CHAMPS study protocol. BMC Public Health.

[52] Brown EL, Ruggiano N, Page TF, Roberts L, Hristidis V, Whiteman KL, Castro J (2016). CareHeroes web and Android™ apps for dementia caregivers: a feasibility study. Res Gerontol Nurs.

[53] Garcia SF, Smith JD, Kallen M, Webster KA, Lyleroehr M, Kircher S, Bass M, Cella D, Penedo FJ (2022). Protocol for a type 2 hybrid effectiveness-implementation study expanding, implementing and evaluating electronic health record-integrated patient-reported symptom monitoring in a multisite cancer centre. BMJ Open.

[54] Foer D, Flores JS, Bates D, Dalal A, Rudin R (2022). Improving mobile health for asthma in a pandemic: patient use of an integrated COVID-19 screener. J Allergy Clin Immunol.

[55] Nouri SS, Avila-Garcia P, Cemballi AG, Sarkar U, Aguilera A, Lyles CR (2019). Assessing mobile phone digital literacy and engagement in user-centered design in a diverse, safety-net population: mixed methods study. JMIR Mhealth Uhealth.

[56] (2022). FACIT-COST. FACIT.org.

[57] Heaney-Huls K, Lobach DF, Kawamoto K, CDSiC Implementation, Adoption, and Scaling Workgroup (2024). Chapter 4. Considerations for assessing PC CDS value. Implementation, Adoption, and Scaling Workgroup: Key Factors and Considerations for Assessing the Value of Patient-Centered Clinical Decision Support.

[58] Zych MM, Bond R, Mulvenna M, Martinez Carracedo J, Bai L, Leigh S (2024). Quality assessment of digital health apps: umbrella review. J Med Internet Res.

[59] Emerson MR, Buckland S, Lawlor MA, Dinkel D, Johnson DJ, Mickles MS, Fok L, Watanabe-Galloway S (2022). Addressing and evaluating health literacy in mHealth: a scoping review. Mhealth.

[60] West L, Mitchell D, Faulkner SD, Bauer B, Brooke N, Priest E (2025). Digital health technologies: learnings and perspectives from a patient engagement stakeholder expectations matrix study. J Med Internet Res.

[61] Yoshida E, Fei S, Bavuso K, Lagor C, Maviglia S (2018). The value of monitoring clinical decision support interventions. Appl Clin Inform.

[62] Wright A, Hickman TT, McEvoy D, Aaron S, Ai A, Andersen JM, Hussain S, Ramoni R, Fiskio J, Sittig DF, Bates DW (2016). Analysis of clinical decision support system malfunctions: a case series and survey. J Am Med Inform Assoc.

[63] Labkoff S, Oladimeji B, Kannry J, Solomonides A, Leftwich R, Koski E, Joseph AL, Lopez-Gonzalez M, Fleisher LA, Nolen K, Dutta S, Levy DR, Price A, Barr PJ, Hron JD, Lin B, Srivastava G, Pastor N, Luque US, Bui TTT, Singh R, Williams T, Weiner MG, Naumann T, Sittig DF, Jackson GP, Quintana Y (2024). Toward a responsible future: recommendations for AI-enabled clinical decision support. J Am Med Inform Assoc.

[64] Grechuta K, Shokouh P, Alhussein A, Müller-Wieland D, Meyerhoff J, Gilbert J, Purushotham S, Rolland C (2024). Benefits of clinical decision support systems for the management of noncommunicable chronic diseases: targeted literature review. Interact J Med Res.

[65] Mobile health app interactive tool. Federal Trade Commission.

[66] SMART health cards and links implementation guide (version 1.0.0 STU1 ballot)—HL7 FHIR R4. Health Level Seven International.

[67] Hamilton CB, Hoens AM, McQuitty S, McKinnon AM, English K, Backman CL, Azimi T, Khodarahmi N, Li LC (2018). Development and pre-testing of the Patient Engagement In Research Scale (PEIRS) to assess the quality of engagement from a patient perspective. PLoS One.

[68] Goodman M, Ackermann N, Pierce K, Bowen D, Thompson V (2021). Development and validation of a brief version of the research engagement survey tool. Int J Environ Res Public Health.

[69] Sawesi S, Rashrash M, Phalakornkule K, Carpenter JS, Jones JF (2016). The impact of information technology on patient engagement and health behavior change: a systematic review of the literature. JMIR Med Inform.

[70] Busse TS, Nitsche J, Kernebeck S, Jux C, Weitz J, Ehlers JP, Bork U (2022). Approaches to improvement of digital health literacy (eHL) in the context of person-centered care. Int J Environ Res Public Health.

[71] Faux-Nightingale A, Philp F, Chadwick D, Singh B, Pandyan A (2022). Available tools to evaluate digital health literacy and engagement with eHealth resources: a scoping review. Heliyon.

[72] Arias López MDP, Ong BA, Borrat Frigola X, Fernández AL, Hicklent RS, Obeles AJT, Rocimo AM, Celi LA (2023). Digital literacy as a new determinant of health: a scoping review. PLOS Digit Health.

[73] Oullet J, Kang J, Girouard A (2021). Cultural and linguistic variables in usability testing: a Canadian evaluation of international students. J Usability Stud.

