# Supplementary material for: Real-World Performance Measurement of Patient-Centered Clinical Decision Support Tools: Qualitative Study
Source: JMIR Form Res. 2026 Sep 16;10:e90000. doi: 10.2196/90000 (PMC13628027; doi:10.2196/90000)
Supplement: Supplementary file 1 [file formative_v10i1e90000_app2.docx]

**ChatGPT Prompts for Measurement Abstraction**

October - November 2023

1. Describe the measures used to evaluate the intervention in the following study: [study text]
2. Describe the pre-implementation, mid-implementation, and post-implementation measures used to evaluate the intervention in the following study: [study text]
3. Describe the measures the research planned to use to evaluate the intervention in the following study protocol: [study text]
4. Describe the types of feedback collected to design the wearable application in the following study: [study text]
5. Describe what feedback researchers sought from patients to inform design of the text messages in the following study: [study text]
