# Supplementary material for: Real-World Performance Measurement of Patient-Centered Clinical Decision Support Tools: Qualitative Study
Source: JMIR Form Res. 2026 Sep 16;10:e90000. doi: 10.2196/90000 (PMC13628027; doi:10.2196/90000)
Supplement: Supplementary file 2 [file formative_v10i1e90000_app3.docx]

**Key Informant Interview Discussion Guide and Protocol**

Overview and Consent

Welcome, and thank you very much for your time today.

*{Interviewer introduces self}*

NORC is under contract with the Agency for Healthcare Research and Quality (AHRQ) at the Department of Health and Human Services to operate the Clinical Decision Support Innovation Collaborative (CDSiC). Launched in 2021, the CDSiC is a community of broad, diverse stakeholders at the forefront of using technology to better support care teams, patients, and caregivers. The CDSiC is working toward healthcare decisions that are driven by both patient-centered and patient-specific information and that align with patient needs, preferences, and values.

This interview will inform a project being conducted to assess how patient-centered clinical decision support (PC CDS) performance is measured in the real world. By PC CDS we mean CDS such as the kind used in your project that either 1) collects and uses new, patient-centric data collected directly from patients (e.g., PGHD and PROs), 2) supports asynchronous and synchronous data display and collection, 3) supports shared decision-making between patients and clinicians, and/or 4) involves reminders sent directly to patients via text, smartphone apps, or patient portals. Given how nascent the field is we are trying to assess what types of measures are being used, what challenges researchers/evaluators experience with measurement, and where might there be opportunities to further develop measures to assess PC CDS performance. By measures we are looking at measures of both structure, process, and outcomes such as those related to patient engagement, usability, and efficiency to name a few.

For today’s call we are hoping to learn more about your measurement experience as well as get your insights regarding gaps and challenges with PC CDS performance measurement generally. We will cover:

1. Your experiences in selecting and using specific measures to assess performance of your intervention
2. Your perspectives on gaps and challenges in PC CDS performance measurement, and
3. Your perspectives on opportunities to improve PC CDS performance measurement.

A few things before we get started:

- The format today will be an informal discussion. We'll ask questions to keep the conversation and demo moving, but we mostly want to hear from you.
- We've scheduled this discussion to last approximately 60 minutes. Your participation is voluntary, and you can conclude the discussion at any time.
- There are no wrong answers to the questions I will ask. You only need to respond to those questions you wish to answer.
- All interview responses will be confidential and stored separately from your contact information. We will not share your contact information or interview responses with anyone outside our study team.
- This is also a good time to emphasize that no reports we may produce throughout the course of this work will include any information identifying you.

I also want to mention that we have another team member on the discussion today taking notes *{introduce note taker}*, and we'd like to make an audio recording of this conversation to help ensure we collect everything. The study team will only use the notes and recordings to write our report. Any written data will not include personally identifiable information, and audio recordings will be destroyed once the project is complete.

- Do you agree to participate in this interview?
- Do we have your permission to record this conversation?

*{Mention that the recording has begun.}*

- Do you have any questions or concerns before we get started?

## Introduction

1. I want to start by having you do a brief introduction of yourself and your AHRQ-funded project: [add title here before interview]. Please introduce yourself and describe the project you did.

## Selection of Project Measures

*In the first set of questions we are hoping to learn more about your experience with selecting and using measures to assess your intervention. We have reviewed your final report to AHRQ and your peer-reviewed publications, so our questions will focus on what we found in our review.*

Below are example questions for a specific project. We updated these questions for each interview.

2. We saw that you conducted a **usability** evaluation of the [app] with patients and usability experts involving think-alouds, a heuristic evaluation, cognitive walkthroughs, and a usability survey. What were some of the challenges or limitations, if any, that you experienced in measuring usability?
   a. *Probe:* Were there any usability measures you wanted to use but either could not or chose not to? If so, are there specific reasons related to measurement that prevented you from collecting and/or assessing this information?
3. We also saw that you conducted an RCT in which you measured some indicators of the **patient’s engagement** in their care, including engagement with their provider and attendance at primary care visits. What were some of the challenges or limitations, if any, that you experienced with these measurements?
   a. *Probe:* Were there any engagement measures you wanted to use but either could not or chose not to? If so, are there specific reasons related to measurement that prevented you from collecting and/or assessing this information?
4. We also saw you that you measured **app use**. What were some of the challenges or limitations, if any, that you experienced with these measurements?
   a. *Probe:* Were there any measures of use you wanted to assess but either could not or chose not to? If so, are there specific reasons related to measurement that prevented you from collecting and/or assessing this information?
5. We also saw that you measured several **health-related outcomes** in the RCT. What were some of the challenges or limitations, if any, that you experienced in measuring outcomes?
   a. *Probe:* To what degree did you feel there were adequate measures to assess the outcomes of your intervention?
   b. *Probe:* Were there any outcome measures you wanted to use but either could not or chose not to? If so, are there specific reasons related to measurement that prevented you from collecting and/or assessing this information?
6. How did you select the measures that you used to assess your intervention? To what degree were you able to identify them in planning the research protocol?
7. Are there any areas of measurement we missed and/or challenges and limitations you want to bring up that we didn’t cover already?

## Gaps in PC CDS Performance Measurement

*The next set of questions concern your perspectives on gaps in PC CDS performance measurement.* *These questions are based on findings from our cross-cutting review of AHRQ-funded PC CDS projects and where we identified gaps in measurement.*

8. What other aspects of your intervention, if any, did you want to measure but were not able to? Why not?
9. In our review, we found that in general projects did not measure the following aspects of PC CDS performance:
   - Technical performance
   - The presentation of information
   - Unintended consequences
   - Costs

Do you have any perspectives on why we do not see measurement in these areas?

Were there any measures in this area you wanted to use but either could not or chose not to? If so, are there specific reasons related to measurement that prevented you from collecting and/or assessing this information?

10. Lastly, we found that few projects measured equity beyond descriptives of participant demographics and socioeconomic information – for example, measuring differences in use or engagement by subgroups. Do you have any perspectives on why we do not see measurement in this area?
    a. *Probe:* Were there any measures in this area you wanted to use but either could not or chose not to? If so, are there specific reasons related to measurement that prevented you from collecting and/or assessing this information?

## Opportunities for PC CDS Performance Measurement

*The final set of questions are intended to gather your perspectives on opportunities to address the challenges and limitations we have been discussing with PC CDS performance measurement, as well as challenges that we identified in our review of AHRQ projects.*

11. Given your work and experience in assessing PC CDS interventions: Are there some measurement areas that you feel should be developed in in the near term so we can conduct more standard assessments of the use of this technology?
    a. If yes, what would specific measures/measurement areas do you feel would be appropriate for this?

12. In the longer term, what measures do you think should be refined/developed?
13. If you could measure anything you wanted to on a project like this, what would that look like?

## Conclusion

Those are all the questions that we had for you today. Is there anything else that you would like to add on this topic?

Thank you again for your time and insights. Please do not hesitate to reach out if you have further questions about this project.

Have a great rest of your day!
