# Supplementary material for: Real-World Performance Measurement of Patient-Centered Clinical Decision Support Tools: Qualitative Study
Source: JMIR Form Res. 2026 Sep 16;10:e90000. doi: 10.2196/90000 (PMC13628027; doi:10.2196/90000)
Supplement: Supplementary file 3 [file formative_v10i1e90000_app4.docx]

**Technical Expert Panel Meeting Discussion Questions**

October 2023 – June 2024

**Overview of Results from Document Review**

1. What is the most informative way to present the overall project characteristics for other researchers or implementers?
2. Do you have any perspectives on why we do not see measurement in the areas of technical performance, information quality, cost, unintended consequences, or equity?
3. Are there other considerations we should keep in mind when interpreting the preliminary results based on project variation?

**TEP Feedback on Preliminary KII Findings**

4. To what extent do these PC CDS measurement challenges resonate with you?
5. What do you think are the main challenges with measuring patient engagement with PC CDS from your experience in the field?

**TEP Feedback on Opportunities to Advance Measurement**

6. To what extent do these “Action Plan” areas to advance PC CDS measurement resonate with you?
7. Are there some measurement areas that you feel should be developed in the near term so we can conduct more standard assessments of PC CDS technology performance?
8. Are you aware of projects/studies already doing work in one of these Action Plan areas to advance PC CDS measurement?
