# Supplementary material for: Real-World Performance Measurement of Patient-Centered Clinical Decision Support Tools: Qualitative Study
Source: JMIR Form Res. 2026 Sep 16;10:e90000. doi: 10.2196/90000 (PMC13628027; doi:10.2196/90000)
Supplement: Supplementary file 4 [file formative_v10i1e90000_app5.docx]

**Table S1.** Study characteristics.

| **No.** | **Study** | **Phase(s) of PC CDS Lifecycle** | **Population and Medical Condition** | **Type of Technology** | **User of Technology** | **Data Collected** |
| --- | --- | --- | --- | --- | --- | --- |
| 1 | Adapting, Scaling, and Spreading an Algorithmic Asthma Mobile Intervention to Promote Patient-Reported Outcomes Within Primary Care Settings (2018-2022) [1] | All | Adults ≥ 18 with asthma | Mobile app | Patient | PROs (Measures related to asthma symptoms) |
| 2 | Improving Diabetes and Depression Self-management Via Adaptive Mobile Messaging (2017-2022) [2] | All | Adults aged 18-75 diagnosed with diabetes and depression | Mobile app; Text messaging program | Patient | PGHD (step counts and self-reported mood) |
| 3 | Development and Evaluation of Patient-Reported Outcome Score Visualization to Improve Their Utilization (PROVIZ) (2019-2021) [3] | All | Adults ≥ 18 with chronic hip/knee pain or osteoarthritis (OA) | Dashboard | Patient, Clinician | PROs (Measures related to pain severity and physical function) |
| 4 | The Wise App Trial for Improving Health Outcomes in PLWH (2016-2022) [4] | All | Adults ≥ 18 diagnosed with HIV taking antiretroviral therapy (ART) | Mobile app | Patient | PGHD (Medication adherence  and physical activity data) |
| 5 | Incorporating Patient-Reported Outcomes into Shared Decision Making with Patients with Osteoarthritis of the Knee (2020-2024) [5] | Implementation and Evaluation | Adults aged 45-89 diagnosed with advanced knee OA | EHR-integrated ML-based decision aid | Patient, Clinician | PROs (Measures related to depression, anxiety, knee OA, health-related quality of life) |
| 6 | Advancing the Collection and Use of Patient-Reported Outcomes Through Health Information Technology (2018-2020) [6] | All | Adults ≥ 65 or actively recovering from injury | Mobile app | Patient, Clinician | PROs (Measures related to physical function, mental health, social health) |
| 7 | The CoolCraig App: Promoting Health by Improving Self-Regulation in Adolescents with ADHD (2018-2021) [7] | Design and Development | Children with Attention deficit hyperactivity disorder (ADHD) | Mobile app; Smartwatch | Patient, Caregiver | PROs (Measures related to mood, self-regulation, self-efficacy)  PGHD (Heart rate, sleep patterns, physical activity) |
| 8 | A Direct-to-Patient Alert for Glycated Hemoglobin Screening Using Prediction Modeling and Mobile Health (mHealth) (2019-2022) [8] | All | Adults at risk for Type 2 Diabetes | Text messaging program | Patient | PGHD (HbA1c screening results) |
| 9 | Examining the Clinical Workflow and Outcomes of Integrating Health Information Technology to Educate and Support Dementia Caregivers (2018-2022) [9] | Implementation and Evaluation | Caregivers ≥ 21 of individuals with Alzheimer’s disease or a related dementia | Mobile app; Web-based platform | Clinician, Caregiver | PROs (Measures related to depression, burden, self-efficacy, satisfaction) |
| 10 | Expansion, Implementation, and Evaluation of Electronic Health Record-Integrated Patient-Reported Symptom Screening in a Comprehensive Cancer Center (2018-2023) [10] | Implementation and Evaluation | Adults diagnosed with cancer | EHR | Patient, Clinician | PROs (Measures related to pain, fatigue, physical function, depression, anxiety, supportive care needs, health-related quality of life) |
| 11 | EHR-based Screening and Intervention for Intimate Partner Violence (2018-2023) [11] | All | Women aged 18-49 at risk of interpersonal violence (IPV) | EHR | Patient | HRSN (IPV screening results) |
| 12 | Integrating Patient-Reported Outcomes into Routine Primary Care: Monitoring Asthma Between Visits (2018-2023) [12] | All | Adults ≥ 18 diagnosed with asthma | Mobile app; Dashboard | Patient, Clinician | PROs (Measures related to symptoms, activity limitations, emotional function, environmental exposures) |
| 13 | An Evaluation of the Spread and Scale of PatientToc™ from Primary Care to Community Pharmacy Practice for the Collection of Patient-Reported Outcomes (2019-2024) [13] | Implementation and Evaluation | General population with nonadherence to medications | Mobile app | Patient, Clinician | PROs (Measures related to medication adherence) |
| 14 | Rheumatology Informatics System for Effectiveness Patient-Reported Outcome (RISE PRO) Dissemination Project (2018-2022) [14] | All | Adults diagnosed with rheumatoid arthritis (RA) | AI-based registry; Dashboard | Patient, Clinician | PROs (Measures related to RA, disease activity, functional status) |
| 15 | Optimizing Acute Post-Operative Dental Pain Management Using New Health Information Technology (2018-2022) [15] | Implementation and Evaluation | Adults ≥ 18 who had undergone endodontic, periodontal, oral surgery or implant procedures | Mobile app; Dashboard; Web-based platform | Patient, Clinician | PROs (Measures related to pain intensity, how pain interfered with daily functions) |
| 16 | Integrating Contextual Factors into Clinical Decision Support to Reduce Contextual Error and Improve Outcomes in Ambulatory Care (2017-2022) [16] | Implementation and Evaluation | General patient population | EHR | Clinician | HRSN (contextual factors impacting medication, appointment, laboratory, and test adherence) |
| 17 | Implementation and Dissemination of 'Gabby,' a Health Information Technology System for Young Women, into Community-Based Clinical Sites (2017-2022) [17] | Implementation and Evaluation | Adult Black women receiving care from community health clinics | AI-based conversational agent; Web-based platform | Patient, Clinician | PROs (Measures related to self-reported pre-conception risks, readiness to address the risk) |
| 18 | Clinical Decision Support for Collaborative Diet Goal Setting in Primary Care (2020-2023) [18] | All | Adults ≥ 18 with a diet-related comorbidity | EHR | Patient, Clinician | PROs (24-hour diet recalls, self-reported diet behavior) |
| 19 | Scalable Decision Support and Shared Decision Making for Lung Cancer Screening (2019-2022) [19] | All | Adults ≥ 55 with smoking history | EHR-integrated app | Patient, Clinician | HRSN (demographic information and smoking risk factors pulled from EHR) |
| 20 | Enabling Shared Decision Making to Reduce Harm from Drug Interactions: An End-to-End Demonstration (2019-2021) [20] | All | Adults at risk of gastrointestinal bleeding due to drug-drug interactions | EHR-integrated app | Patient, Clinician | Patient preferences (Preferences related to how pain is treated, types of medications) |

## **References**

1. Jariwala SP, Rastogi D, Dlugacz Y, et al. Adapting, scaling, and spreading an algorithmic asthma mobile intervention to promote patient-reported outcomes within primary care settings. Final Report. Prepared by Albert Einstein College of Medicine under Grant No. R18 HS025645. Rockville, MD: Agency for Healthcare Research and Quality; 2022. Accessed June 23, 2025.
2. Aguilera A, Lyles C, Garcia F, et al. Improving Diabetes and Depression Self-Management via Adaptive Mobile Messaging (DIAMANTE). Final Report. Prepared by the University of California, Berkeley and University of California, San Francisco under Grant No. R01 HS025429. Rockville, MD: Agency for Healthcare Research and Quality; 2022. Accessed June 23, 2025. https://digital.ahrq.gov/sites/default/files/docs/citation/r01hs025429-aguilera-final-report-2022.pdf
3. Gold H, Bertini E. Development and Evaluation of Patient-Reported Outcome Score Visualization to Improve Their Utilization (PROVIZ). Final Report. Prepared by the New York University School of Medicine under Grant No. R21 HS027228. Rockville, MD: Agency for Healthcare Research and Quality; 2021. Accessed June 23, 2025. https://digital.ahrq.gov/sites/default/files/docs/citation/r21hs027228-gold-final-report-2021.pdf
4. Agency for Healthcare Research and Quality. WISE app trial: improving health outcomes in people living with HIV. Accessed June 23, 2025. <https://digital.ahrq.gov/ahrq-funded-projects/wise-app-trial-improving-health-outcomes-plwh>
5. Lin E, Uhler LM, Finley EP, et al. Incorporating patient-reported outcomes into shared decision-making in the management of patients with osteoarthritis of the knee: a hybrid effectiveness-implementation study protocol. BMJ Open. 2022;12(2):e055933. Published 2022 Feb 21. doi:10.1136/bmjopen-2021-055933
6. Agency for Healthcare Research and Quality. Advancing the collection and use of patient-reported outcomes through health information technology. Accessed June 23, 2025. <https://digital.ahrq.gov/ahrq-funded-projects/advancing-collection-and-use-patient-reported-outcomes-through-health>
7. Lakes KD, Hayes GR, Schuck SEB, Cibrian F, Nelson M. The CoolCraig app: promoting health by improving self-regulation in adolescents with ADHD. Final Report. Prepared by the University of California, Riverside under Grant No. R21 HS026058. Rockville, MD: Agency for Healthcare Research and Quality; 2022. Accessed June 23, 2025. https://digital.ahrq.gov/sites/default/files/docs/citation/r21hs026058-lakes-final-report-2022.pdf
8. Wells BJ, Sandberg JC, Miller DP Jr, McClain DA. A direct-to-patient alert for glycated hemoglobin screening using prediction modeling and mobile health (mHealth). Final Report. Prepared by Wake Forest University School of Medicine under Grant No. R21 HS026803. Rockville, MD: Agency for Healthcare Research and Quality; 2022. Accessed June 23, 2025. https://digital.ahrq.gov/sites/default/files/docs/citation/r21hs026803-wells-final-report-2022.pdf
9. Agency for Healthcare Research and Quality. Examining clinical workflow and outcomes from integrating health information technology. Accessed June 23, 2025. <https://digital.ahrq.gov/ahrq-funded-projects/examining-clinical-workflow-and-outcomes-integrating-health-information>
10. Garcia SF, Penedo FJ, Bass M, David Cella, Sheetal Kircher, Justin Dean Smith, Kimberly A. Webster. Expansion, implementation, and evaluation of electronic health record-integrated patient-reported symptom screening in a comprehensive cancer center. Final Report. Prepared by Northwestern University Feinberg School of Medicine, Department of Medical Social Sciences under Grant No. R18 HS026170. Chicago, IL: Agency for Healthcare Research and Quality; 2022. Accessed June 23, 2025. https://digital.ahrq.gov/sites/default/files/docs/citation/r18hs025131-jack-final-report-2022.pdf
11. Agency for Healthcare Research and Quality. EHR-based screening and intervention for intimate partner violence. Accessed June 23, 2025. <https://digital.ahrq.gov/ahrq-funded-projects/ehr-based-screening-and-intervention-intimate-partner-violence>
12. Agency for Healthcare Research and Quality. Integrating patient-reported outcomes into routine primary care for monitoring asthma. Accessed June 23, 2025. <https://digital.ahrq.gov/ahrq-funded-projects/integrating-patient-reported-outcomes-routine-primary-care-monitoring-asthma>
13. Snyder ME, Chewning B, Kreling D, et al. An evaluation of the spread and scale of PatientToc™ from primary care to community pharmacy practice for the collection of patient-reported outcomes: a study protocol. *Res Social Adm Pharm*. 2021;17(2):466-474. doi:10.1016/j.sapharm.2020.03.019
14. Agency for Healthcare Research and Quality. Rheumatology Informatics System for Effectiveness–Patient-Reported Outcome (RISE-PRO). Accessed June 23, 2025. <https://digital.ahrq.gov/ahrq-funded-projects/rheumatology-informatics-system-effectiveness-patient-reported-outcome-rise-pro>
15. Kalenderian E, Walji M, White JM, Yansane AI, Skourtes N, Mullins J, Brandon R, Holmes D. Optimizing acute post-operative dental pain management using new health information technology. University of California, San Francisco and The University of Texas School of Dentistry at Houston; 2022. Agency for Healthcare Research and Quality. Grant No. U18HS026135. Available at: https://digital.ahrq.gov/sites/default/files/docs/citation/u18hs026135-white-final-report-2022.pdf
16. Weiner SJ, Schwartz A, Weaver F, et al. Integrating contextual factors into clinical decision support to reduce contextual error, improve outcomes, and reduce misuse, overuse, and underuse of outpatient primary care. Agency for Healthcare Research and Quality. Accessed June 10, 2025. https://digital.ahrq.gov/sites/default/files/docs/citation/r01hs025374-weiner-final-report-2022.pdf
17. Jack BW, Paasche-Orlow MK, Doros G. Implementation and dissemination of 'Gabby,' a health information technology system for young women, into community-based clinical sites. Agency for Healthcare Research and Quality. Accessed June 23, 2025. https://digital.ahrq.gov/sites/default/files/docs/citation/r18hs025131-jack-final-report-2022.pdf
18. Agency for Healthcare Research and Quality. Clinical decision support for collaborative diet goal setting in primary care. Accessed June 23, 2025. <https://digital.ahrq.gov/ahrq-funded-projects/clinical-decision-support-collaborative-diet-goal-setting-primary-care>
19. Kawamoto K, Caverly T, Del Fiol G, Fagerlin A, Flynn M, Hess R, Kukhareva P, Martin D, Ranade P, Reddy C, Weir C, Zhang Y. Scalable decision support and shared decision making for lung cancer screening. University of Utah; 2022. Agency for Healthcare Research and Quality. Grant No. R18HS026198. Available at: https://digital.ahrq.gov/sites/default/files/docs/citation/r18hs026198-kawamoto-final-report-2022.pdf
20. Agency for Healthcare Research and Quality. Enabling shared decision making to reduce harm from drug interactions: an end-to-end approach. Accessed June 23, 2025. <https://digital.ahrq.gov/ahrq-funded-projects/enabling-shared-decision-making-reduce-harm-drug-interactions-end-end>
